# Spanish consensus on managing pregnancy in women with Gaucher disease

**DOI:** 10.1186/s13023-025-03684-6

**Published:** 2025-03-28

**Authors:** Enrique J. Calderón, Alicia Rodríguez-Fernández, Irene Calderón-Baturone, Rafael Aporta-Rodríguez, Francisco J. del Castillo, Lutgardo García-Díaz, Antonio González-Meneses, María Lourdes Hermosín-Ramos, Raquel Yahyaoui, Ignacio Marín-León

**Affiliations:** 1 Instituto de Biomedicina de Sevilla, Centro de Investigación Biomédica en Red de Epidemiología y Salud Pública (CIBERESP), Hospital Universitario Virgen del Rocío/Consejo Superior de Investigaciones Científicas/Universidad de Sevilla, Seville, Spain; 2 Departamento de Medicina, Facultad de Medicina, Universidad de Sevilla, Seville, Spain; 3 Virgen Macarena University Hospital, Hematology, Seville, Spain; 4 Ceuta General Hospital, Hematology, Ceuta, Spain; 5 Hospital Universitario Ramón y Cajal-IRYCIS, Genetics, CIBERER U728, Madrid, Spain; 6 Fuerteventura General Hospital, Gynecology, Fuerteventura Spain; 7 Virgen del Rocio University Hospital, Gynecology, Seville, Spain; 8 Departamento de Farmacología, Pediatría y Radiología, Seville, Spain; 9 Virgen del Rocio University Hospital, Pediatric, Seville, Spain; 10 Jerez General Hospital, Hematology, Jerez, Spain; 11 Málaga Regional University Hospital, Unit of Inherited Metabolic Disorders. IBIMA-Plataforma BIONAND, Málaga, Spain; 12 Fundación Enebro, Seville, Spain

**Keywords:** Gaucher disease, Pregnancy, Guideline

## Abstract

Gaucher disease can have effects on the development of pregnancy, childbirth, and lactation, with impact on health of both the mother and the newborn. Management of pregnancies in Gaucher patients is further complicated by using of enzyme replacement therapy. Unfortunately, the available scientific evidence is not conclusive because there are not proper clinical trials on this issue. The aim of this work was to establish a management guide to address the main clinical problems before, during and after pregnancy and to provide key information to healthcare professionals, patients, and families. GRADE methodology to evaluate the quality of scientific evidence and develop recommendations was incorporated to elaborate this guide. For final recommendations, a structured consensus 2-round process was carried out using the Delphi method with a Gaucher expert panel. After this process, nine recommendations were elaborated related to pre-pregnant status and genetic counseling and for management during pregnancy, seven related to childbirth, and eight focused on management after delivery and breastfeeding. Regarding the quality of the evidence, values and preferences of patients were also considered. A consensus guide to define and standardize pregnancy management in Gaucher disease considering the best available evidence, complemented by experts’ opinions, could be a relevant tool to help patients, nurses, midwives and physicians with little experience in Gaucher disease who do not have access to guidance from Gaucher disease treatment centers of excellence.

## Background

Gaucher disease (GD: MIM#230800)) is an inherited metabolic disorder resulting from mutations in the gene that encodes the enzyme glucocerebrosidase (acid β-glucosidase; EC 3.2.1.45) that produces a deficient activity of β -glucocerebrosidase and the subsequent accumulation of the glycolipid glucosylceramide in cells of the monocyte-macrophage system [1]. The non–neuropathic form, named type 1, is the most common lysosomal storage disorder and has a highly variable phenotypical range from total asymptomatic to severe disease with splenomegaly (with associated hypersplenism and thrombocytopenia, anemia and leucopenia), hepatomegaly, and abnormal coagulation and often bone disease including bone pain, bone crises, abnormal bone (re)modelling, osteopenia, osteonecrosis and pathological fractures, and in some cases lung involvement [2]. In type 1 Gaucher disease (GD), girls reach reproductive age and therefore her reproductive health can be affected, including fertility, gravidity, pregnancy, delivery, postpartum complications, and lactation, but the available information about these issues is scarce and comes mainly from case series reports [3–11].

Pregnancy, labor, and delivery in women with type 1 Gaucher disease can represent a problem for themselves and for clinicians due to abnormal coagulation and multiorgan affectation. During pregnancy there is a reduction in hemoglobin levels and approximately be-tween 5–11% of all pregnancies have gestational thrombocytopenia, which does not confer an increased risk of maternal bleeding [12, 13]. However, in pregnant women with GD, these alterations may worsen above those normally associated with pregnancy, and excessive bleeding may complicate pregnancy, delivery, and postpartum, mainly if these women have impaired platelet function [4, 14–16]. Visceromegaly may interfere with normal fetal growth and condition mode of delivery [14]. Orthopedic complications related to GD could also affect the mode of delivery [17]. The greatest demand for calcium for the female skeleton occurs during pregnancy and lactation increasing potentially the risk of bone crises, osteopenia, osteonecrosis, and fractures [4, 14, 18].

Moreover, pregnancy could exacerbate the course of GD or induce the appearance of new manifestations of the disease [6]. The evolution of GD during pregnancy is diverse and related mainly to the severity of the disease and therapy control at the beginning of the pregnancy [14]. Women with GD can experience an increase in volume of hepatosplenomegaly, bone crises, osteoporosis, anemia, or worsening of thrombocytopenia [4, 14]. Furthermore, during pregnancy, symptoms can arise that lead to the diagnosis of Gaucher disease in previously undiagnosed women [14].

Today, there are two specific ways to treat GD: enzyme replacement therapy (ERT) with several available recombinant enzymes, velaglucerase alfa, imiglucerase and taliglucerase alfa, and substrate reduction therapy (SRT) with two different drugs, eliglustat and miglustat. However, not all of them can be used during pregnancy or lactation [19].

The management of women with Gaucher disease type 1 before becoming pregnant, during pregnancy, and after delivery is a major challenge for all physicians caring for these patients. However, there are no clinical trials on this issue, both because of the number of patients that would be necessary and because of the ethical problem that the use of placebo would pose. In fact, only a few outdated consensus recommendations or expert opinion have been published to support decision-making and guide treatment of these patients and there are no recommendations about in vitro fertilization and preimplantation genetic screening for women with GD [20–23].

The purpose of this article is to establish management guidance for the GD-pregnancy relationship to address the main clinical problems with respect to (1) genetic counseling, (2) treatment before, during, and after pregnancy, (3) approach to childbirth and breastfeeding, (4) prevention of hematologic and bone complications in mother and child, and (5) key information for patients and their families.

## Materials and methods

The methodology for this project is based on the 2016 Methodological manual for developing clinical practice guidelines of the Spanish National Health System and incorporates the Grading of Recommendations Assessment, Development and Evaluation (GRADE) methodology in the assessment of the scientific evidence and strength of the recommendations [24, 25].

To define the objective of the consensus, the clinicians’ and patients’ needs for decision advice was explored with a survey asking for the main disease-specific issues that concerned them. The development group designed a map of the healthcare process for pregnancy in GD, structuring the process into 4 steps or chapters (pre-pregnancy and genetic counseling; pregnancy; childbirth, and post-pregnancy and breastfeeding). For each chapter, the group specified the potential issues that clinicians and patients could establish on each part of the healthcare process, addressing mutually exclusive aspects in each issue, resulting in 56 PICO (population, intervention, comparison, outcome) items.

### Evidence search

Studies published between January 2000 and April 2021 were searched. The search strategy was not restricted by language or article type.

For the PubMed search strategy, the key words ‘Gaucher disease’ AND ‘pregnancy’ OR ‘fertility’ OR ‘childbirth’ OR ‘labor’ OR ‘genetic counseling’ OR ‘baby feeding’ OR ‘mother outcomes’ OR ‘child outcomes’ were used. The same strategy was executed in the other databases using the appropriate controlled vocabulary. The following electronic databases were also explored for primary studies: Scopus, EBSCO, Academic Search Complete, CINAHL, Biomedical Reference.

Due to the scarcity of bibliography available, other resources including grey literature from different sources and hand-searching of those high-yield journals and conference proceedings that have not already been hand-searched for opinion articles, guidelines, consensus, and the references supporting those documents were also reviewed.

### Studies selection

The studies found through the search strategy were screened by two reviewers (EJC, IML), who reviewed all of them and given the few studies collected, with the sole criterion of excluding the repeated articles.

### Data extraction

Three authors (EJC, IML, ARF) independently classified the studies by type and tried to undertake data extraction to get the evidence standard of care available for our four clinical scenarios proposed.

### Consensus process

A consensus was reached among nine experienced GD experts from 6 specialties related to Gaucher disease care (hematologists, internists, geneticist, biochemist, pediatrician, gynecologist) and a midwife. For the recommendation decision we used a structured 2 round Delphi-RAND consensus Method. This method is a structured communication technique, originally developed as a systematic, interactive forecasting method that relies on a panel of experts. The process involves multiple rounds of anonymous surveys. After each round, a facilitator provides a summary of the experts’ forecasts and reasons for their judgments. Experts are encouraged to revise their earlier answers in light of the replies of other members of their panel. This iterative process is repeated until the group reaches a consensus. Key features of the Delphi-RAND method include anonymity, controlled feedback, and statistical aggregation of group responses [26].

In the first round, members of the expert panel were asked to rate agreement with each statement, on a 9-point Likert scale, ranging from strongly agree to strongly disagree (higher score corresponding with agreement). No attempts were made to force consensus.

The 2-round process was designed to determine “whether discrepant ratings are due to real clinical disagreement over the use of the procedure (‘real’ disagreement) or to fatigue or misunderstanding (‘artifactual’ disagreement), and to address any remaining significant discord and obtain consensus. For that purpose, the outcome of the first round was analyzed by the facilitator/expert in the Delphi method (IM), and the results were reviewed by the coordinators (EC, AR), which revised some statements that presented syntax/communication issues. A summary report describing round 1 group responses was sent to the nine GD experts included in the panel via email. Statements ranked with a median score of 7 or higher were considered “strong” agreement (51.3%) and, thus, complete, while median scores ranked 4 to 6 were considered “uncertain” agreement (27,6%), and median scores ranked 1 to 3 were considered “disagreement (21%). Statements that achieved a median score of 7 or higher in round 1 but had some rankings from individual experts from 1 to 6 were considered to vote on in the second round. The statements that did not reach agreement or disagreement were evaluated and re-proposed with an explicative text during the second round of Delphi.

Values and preferences of patients were also considered through a brainstorming meeting with patient associations.

Three (EC, AR, IM) members of the panel wrote a draft of the recommendations with the results of Delphi consensus. Panel members were asked to review the recommendations and give their agreement with a ‘yes / no’ response (“yes” indicating agreement with the revised standard and inclusion in the consensus recommendations and ‘no’ indicating exclusion from the consensus recommendations). Minor editing (e.g., grammar and structure) was allowed, but no change to intent was made. Finally, the final draft of the recommendations was approved by all panel members.

## Results

Very little evidence addressing GD and pregnancy was founded. Bibliography search provided 36 references that were classified as 3 prospective observational series, with a total of 64 pregnancies; 6 retrospective observational series with a total of 505 pregnancies; 9 case records; and 8 narrative reviews and opinion article mainly focused on pre-pregnancy and genetic counseling. The quality of evidence was low due to the heterogeneity of publication dates, sample sizes, treatments, and outcome data reported, with agreement among reviewers.

Thirty-two recommendations were drafted, 9 for pre-pregnant and genetic counseling, 9 for the pregnancy period, 7 for childbirth and 7 for postpartum and breastfeeding. For three PICOs the panel did not agree on a recommendation (fertility issues in GD patients, delays in the menarche of women with GD, and specific role of midwives in the process of care). The strength of recommendations agreement was 24 strong (recommendation can apply to most patients in most circumstances) and 8 weak (the best course of action may differ depending on circumstances or patient or society values. Other alternatives may be equally reasonable).

The questions related to recommendations organized by four steps in the care process, along with the strength of their support between the panel members (in brackets), are shown below.

### Panel recommendations

#### Actions before pregnancy

1.1 Should all patients with Gaucher’s disease (GD) who wish to bear children be offered genetic preconception counseling? Yes (Strong Agreement).
1.2 Can assisted reproduction techniques be used in patients with GD and fertility problems? Yes (Strong Agreement).

Furthermore, assisted reproduction techniques are compatible with enzyme re-placement therapy (ERT): Yes (Strong Agreement).

Comment: Because alterations in the function or number of platelets may affect in vitro fertilization (IVF), the panel recommends a personalized approach to cope with all the determinants of GD status in each patient, together with the number of platelets, or their functionality when this is greater than 100,000 /mm3, which is when these functionality tests are reliable. It is also needed to assess risks from the use of acetyl salicylic acid (ASA), to improve reproductive outcomes and avoid the risk of thrombosis by IVF. So that the risk/benefit balance the risk of fetal loss or ovarian puncture bleeding, and a shared decision on assisted reproduction could be made.

1.3 In the event that the spouse is a GD carrier or the donor is unknown, should a pre-implantation genetic diagnosis be offered in an IVF-affected woman?: Yes. (Weak Agreement). Comment: If a GD carrier is detected, it should be required to investigate possible pseudo-deficiencies of the enzyme glucocerebrosidase. Even in Ashkenazi Jews it can be done when the Gaucher situation of the spouse is unknown, to facilitate the detection of specific mutations in the embryo. However, because the risk/benefit balance regarding fetal loss and ovarian puncture bleeding is unknown, a process of shared decision-making with the patient and family is mandatory as well as planned annotation of the outcome data in an active GD pregnancy sub-registry.
1.4 From the perspective of the patient’s health, is there ever a reason for a physician to discourage pregnancy in a woman with GD?: Yes (Strong Agreement).

Comment: There are no eugenic conditions, but the evolution of the patient’s disease can condition a pregnancy, especially due to moderate to severe pulmonary hypertension (PH) that this is very rare, and the risk is largely restricted to GD1 women who have undergone a splenectomy or GD type 3 patients with known lung disease. There is a strong agreement that neither anemia (Hb < 10 g/dl), nor thrombocytopenia (< 70,000 dl), nor hepatomegaly or splenomegaly (> 2 times normal), nor osteoporosis are reason to discourage pregnancy. Although it is important to have good communication with the patient about possible risks and concomitant need to act on those risks, such as the need of calcium, vitamin D, or others. In addition, there are concurrent illnesses not directly related to GD (e.g., uncontrolled severe arterial hypertension, brittle diabetes mellitus, systemic lupus erythematosus, psychiatric problems, etc.) when pregnancy could be discouraged.

1.5 Should a comprehensive GD patients assessment have been performed prior to a planned pregnancy? Yes. (Strong Agreement).

Comments: The panel recommends 3–6 months before pregnancy as a way to have time to correct GD problems during pregnancy. That assessment should include in addition to the routine controls of a patient with GD, a bone densitometry and cardiac ultrasound to rule out PH, given the great marginal benefit de-rived from its detection, despite it is an uncommon complication. In case of inconclusive cardiac ultrasound or borderline for PH, the reference technique is right heart catheterization. This is an invasive technique, and risk/benefit should be assessed and the decision to do should be made in agreement with the patient.

1.6 How long before conception should women with GD stop bisphosphonates treatment? Bisphosphonates should be suspended at least 6 months before pregnancy, or longer for those long-lasting bisphosphonates, which should be suspended before the end of the period of intended effect. (Strong Agreement).
1.7 In splenectomized patients who want to plan a pregnancy some special measures should be taken into account? Yes. (Strong Agreement). They should be vaccinated against pneumococcus, meningococcus, *Haemophilus influenzae* and influenza. Centers for disease control and prevention (CDC) recommend that all adults without spleen to get a primary series of COVID-19 vaccine plus booster doses when eligible [27]. In addition, a cardiac ultrasound has to do to rule out PH.
1.8 Pregnancy and ERT: GD patients on ERT should not suspend or modify such treatment for their pregnancy even in the first trimester.

Asymptomatic GD patients without ERT after a comprehensive GD evaluation should not start treatment before pregnancy.

GD patients with mild symptoms without ERT should start treatment before pregnancy. At doses of 60 units/kg every two weeks. (Strong Agreement).

1.9 Pregnancy and substrate reduction therapy (SRT): Patients on treatment with Miglustat or Eliglustat should discontinue such treatment at least 3 months before pregnancy. And they must change to ERT before pregnancy. (Strong Agreement).

#### Gaucher disease management during pregnancy

2.1 For a pregnant GD woman, is the standard recommended control care designed for women with high-risk pregnancies sufficiently specific for pregnancy in a woman with GD? No. (Strong Agreement).

A specific program adapted to the particular circumstances of each pregnant Gaucher patient, led by an expert multidisciplinary team, is required.

2.2 What specific check-ups should be carried out during the first trimester of pregnancy in a GD patient? Measurement of lysoGb1 and/or chitotriosidase and/or, calcium, vitamin D and vitamin B12 levels. (Strong Agreement).
2.3 Can patients with GD who are pregnant with IVF receive treatment with Aspirin? Yes. (Weak Agreement). Conditioned to thrombocytopenia and normal platelet function.
2.4 Should preventive supplements be added to pregnant women with GD? Yes. (Strong Agreement).

Calcium and vitamin D should be added. Regarding vitamin B12, preventive provision should be made to ensure the daily dose required before and during pregnancy due to its special risk of anemia and neuropathy.

In addition, when low B12 levels were detected at the time of diagnosis of pregnancy (up to week 9), B12 should be provided at therapeutic doses.

2.5 What specific routine check-ups should be carried out during the second and third trimesters of pregnancy in the GD woman? Measurement of lysoGb1 and/or chitotriosidase and/or lysoGb1, calcium levels, and vitamin D. (Strong Agreement).
2.6 Are other routine pregnancy check-ups needed during the third trimester in patients with GD? Yes. (Strong Agreement). A study of platelet function and ultrasound evaluation of liver and spleen sizes should be performed if they were not performed before during pregnancy control.

Comments: Multiple methods of in vitro platelet function evaluation, such as platelet function analyzer (PFA)-100/200, platelet aggregometry, flow cytometry, and cone and platelet analyzer, can be used to assess primary hemostasis. PFA-100/200 is widely used to assess platelet function and has been proposed to assess the risk of presurgical bleeding as a replacement for the ‘skin bleeding time’ [28]. However, this method has not been tested on GD. There is only one study in patients with GD using the cone platelet (let) analyzer [29]. In this study, platelet aggregation defect was not associated with mucosal bleeding and reduced platelet adhesion was [29]. However, there is no specific information for either method in pregnant women with GD.

Although in case of cesarean delivery, the low transversa incision is recommended, an ultrasound study in the last trimester is recommended to document or rule out hepatosplenomegaly (Weak Agreement).

2.7 Should magnetic resonance imaging tests be performed on pregnant GD patients suffering from bone crises or exacerbations of bone pathology? Yes. (Strong Agreement).
2.8 During pregnancy an exacerbation of the GD should lead to perform a dose adjustment of enzyme replacement treatment?” Yes, (Strong Agreement).
2.9 Should prenatal genetic diagnosis be offered in pregnant women with GD? Yes, when the partner is a carrier or in IVF when the donor is unknown. Therefore, a pre-conceptional genetic study of carrier status in couples is always recommended to identify embryos that only will carry one GD mutation or who will have two mutations and therefore will be affected by GD and also to be able to decide depending on the type and risk of the kind of mutations. (Weak Agreement).

#### Gaucher disease care during childbirth

3.1 Where should the delivery of a pregnant woman with GD take place? In a hospital with GD expertise. (Strong Agreement).

Comment: GD requires anticipating any possible complications, such as immediate access to a blood bank.

3.2 Should cesarean delivery be recommended for patients with GD? No. (Strong Agreement).
3.3 Is vaginal delivery possible in GD patients with hip prosthesis? Yes. (Weak Agreement).

Comment: with traumatology and rehabilitation planned support.

3.4 Does the presence of hepatosplenomegaly prevent to perform a cesarean section if necessary? No. (Strong Agreement).

In case of cesarean delivery, the low transversal incision is recommended. (See recommendation 2.6).

3.5 What would contraindicate epidural anesthesia in a pregnant woman with GD? Presence of platelet count less than 70,000/mm3 or impaired platelet function. (Strong Agreement).
3.6 Do pregnant women with GD have a higher risk of miscarriage? Uncertain. Comment: The available literature is inconclusive due to biases and inconsistent re-sults, suggesting the need for further research comparing with non-GD pregnant sample.
3.7 Do pregnant GD women have a higher risk of bleeding or postpartum infections? Yes. (Strong Agreement).

#### Postpartum care

4.1 Should the newborn (NB) undergo genetic testing for GD? No. (Strong Agreement).

If it is not known whether the father may be a carrier of a Gaucher mutation, the newborn should be given an enzymatic dried drop test (neonatal screening) instead of a genetic test. However, sporadically there may be false negatives from the dry drop, in which case if the clinical suspicion is high, the test could be repeated, or a genetic study could be considered.

4.2 Does a newborn without Gaucher disease born to a mother with the disease need any different control than usual NBs? Yes. (Weak Agreement).

Only in case the mother has presented some complication during pregnancy. Otherwise, the child only needs the standard care in the NBs.

4.3 Should breastfeeding be recommended for GD mothers? For how long? Yes, up to 6 months. (Strong Agreement).

Although in case of severe osteoporosis or uncontrolled GD status of the mother it is suggested to avoid breastfeeding. (Weak Agreement).

4.4 Should ERT be maintained during breastfeeding? At what dose? Yes, at the same previous dose. (Strong Agreement).
4.5 Should GD nursing mothers receive vitamin D and calcium supplements? Yes. (Strong Agreement).
4.6 Can GD nursing mothers be treated with bisphosphonates? No. (Strong Agreement).
4.7 Can GD nursing mothers be treated with Miglustat or Eliglustat? No. (Strong Agreement).

The strength of the recommendations is summarized in Tables 1, 2, 3 and 4.

**Table 1:** Recommendations to prepare for pregnancy

Recommendation	Strength
All patients, men or women, who wish to bear children should receive a genetic preconception counseling	Strong
Women with GD and fertility problems can use assisted reproduction techniques	Strong
Pre-implantation genetic diagnosis in cases of in vitro fertilization should do when the spouse is a GD carrier in all cases, or the donor is unknown in Ashkenazi Jews	Weak
Pregnancy is discouraged in case of moderate-severe pulmonary hypertension	Strong
A comprehensive GD women assessment must be performed 3–6 months prior to a planned pregnancy	Strong
Women under bisphosphonates treatment must stop it at least 6 months before pregnancy, or longer for those long-lasting bisphosphonates	Strong
Splenectomized women who want to plan a pregnancy must vaccinate against pneumococcus, meningococcus, Haemophilus influenzae, and flu	Strong
Asymptomatic GD women without enzyme replacement therapy do not need start treatment before pregnancy	Strong
Women with GD with mild symptoms without specific treatment should start enzyme replacement therapy before pregnancy	Strong
Patients on treatment with Miglustat or Eliglustat must switch such treatment to enzyme replacement therapy at least 3 months before pregnancy	Strong

**Table 2:** Recommendations during pregnancy

Recommendation	Strength
The standard recommended control care for pregnant women is not enough for GD women which need a specific program adapted to the circumstances of each case, led by an expert multidisciplinary team	Strong
During the first trimester of pregnancy of chitotriosidase and/or lysoGb1, calcium, vitamin D and vitamin B12 levels must be measurement	Strong
Women with GD who are pregnant through in vitro fertilization can receive treatment with Aspirin if they have normal platelet function and have not significative thrombocytopenia	Weak
Calcium and vitamin D must be added during pregnancy, and a preventive supply of vitamin B12 must be made to ensure the required daily dose or to be administered in therapeutic doses if low levels are detected in laboratory controls	Strong
During the second and third trimesters of pregnancy of chitotriosidase and/or lysoGb1, calcium and vitamin D levels must be measurement	Strong
A study of platelet function must do during the third trimester	Strong
An ultrasound study in the last trimester is recommended to document or rule out hepatosplenomegaly	Weak
In case of bone crises or exacerbations of bone pathology during pregnancy, a magnetic resonance imaging study must be performed	Strong
In case of exacerbations of GD during pregnancy, a dose adjustment of treatment must be performed.	Strong
Prenatal genetic diagnosis might be offered in pregnant women with GD when the partner is a carrier of GD or in the case of in vitro fertilization when the donor is unknown.	Weak

**Table 3:** Recommendations during childbirth

Recommendation	Strength
The delivery of a pregnant woman with GD must occur in a hospital that has immediate access to a blood bank	Strong
Cesarean delivery is not necessary for patients with GD	Strong
Vaginal delivery is possible in GD patients with a hip prosthesis with planned traumatology and rehabilitation support	Weak
If necessary, the presence of hepato-splenomegaly does not prevent the operation of a cesarean section, but the low transversal incision is recommended	Strong
The presence of a platelet counts less than 70,000/mm3 or impaired platelet function contradicts epidural anesthesia in a pregnant woman with GD	Strong
Bleeding and infections should be monitored especially in pregnant women with GD during their delivery	Strong

**Table 4:** Recommendations after delivery

Recommendation	Strength
If an enzymatic dry drop test (neonatal screening) can be offered, the newborn does not need a genetic test for GD. Sporadically there may be false negatives from the dry drop, in which case if the clinical suspicion is high, the test could be repeated, or a genetic study could be considered	Strong
A newborn of a GD mother only needs standard care for newborns unless the mother has presented some complication during pregnancy	Weak
Breastfeeding must be recommended to GD mothers up to 6 months unless she has severe osteoporosis or an uncontrolled status of her GD	Weak
Enzyme replacement therapy must be maintained at the same previous dose during breastfeeding	Strong
Nursing mothers with GD must receive vitamin D and calcium supplements	Strong
Nursing mothers with GD cannot be treated with bisphosphonates	Strong
Nursing mothers with GD cannot be treated with Miglustat or Eliglustat	Strong

### Clinical questions not addressed in the consensus process and that require further research

Do patients with GD (women and men) have fertility problems greater than the non-affected population?

Do patients with GD have delayed menarche?

What is the role of midwives in the management of childbirth of a patient with GD?.

## Conclusions

Women with Gaucher disease type 1 are usually diagnosed just before or during their reproductive age and many alterations related to the disease can affect reproduction capacity or the normal development of pregnancy and delivery [2, 14]. Today, we have different options for effective treatment of Gaucher disease type 1, but we lack specific guidelines on their use in pregnant women with GD [19].

In this work, a management guide that includes 32 recommendations to support the decision-making of physicians caring for women with type 1 GD who want to become pregnant or are pregnant has been developed through a rigorous and reproducible methodology.

For easy use of this guide, recommendations have been grouped within the four principal areas of action or steps identified by the expert panel where it is necessary to make decisions when a woman decides deciding to have children: before conception, during pregnancy, around the partum, and during lactation.

It is hoped that a consensus guidance, such as this, to define and standardized the management of pregnancy in GD in light of the best available evidence, complemented by the opinions of a panel of experts, could be a relevant tool to help patients, nurses, midwives and doctors with little experience in Gaucher disease.

## Data Availability

Data sharing not applicable to this article as no datasets were generated or analysed during the current study.
